# Sex-dependent effects on gut microbiota regulate hepatic carcinogenic outcomes

**DOI:** 10.1038/srep45232

**Published:** 2017-03-27

**Authors:** Guoxiang Xie, Xiaoning Wang, Aihua Zhao, Jingyu Yan, Wenlian Chen, Runqiu Jiang, Junfang Ji, Fengjie Huang, Yunjing Zhang, Sha Lei, Kun Ge, Xiaojiao Zheng, Cynthia Rajani, Rosanna A. Alegado, Jiajian Liu, Ping Liu, Jeremy Nicholson, Wei Jia

**Affiliations:** 1 Shanghai Key Laboratory of Diabetes Mellitus and Center for Translational Medicine, Shanghai Jiao Tong University Affiliated Sixth People’s Hospital, Shanghai 200233, China; 2 University of Hawaii Cancer Center, Honolulu, Hawaii 96813, USA; 3 E-institute of Shanghai Municipal Education Committee, Shanghai University of Traditional Chinese Medicine, Shanghai 201203, China; 4 Key Laboratory of Liver and Kidney Diseases (Ministry of Education), Institute of Liver Diseases, Shuguang Hospital, Shanghai University of Traditional Chinese Medicine, Shanghai 201204, China; 5 Department of Oceanography, University of Hawaii at Mānoa, Honolulu, Hawaii 96822, USA; 6 Biomolecular Medicine, Department of Surgery and Cancer, Faculty of Medicine, Imperial College, London SW7 2AZ, United Kingdom

## Abstract

Emerging evidence points to a strong association between sex and gut microbiota, bile acids (BAs), and gastrointestinal cancers. Here, we investigated the mechanistic link between microbiota and hepatocellular carcinogenesis using a streptozotocin-high fat diet (STZ-HFD) induced nonalcoholic steatohepatitis-hepatocellular carcinoma (NASH-HCC) murine model and compared results for both sexes. STZ-HFD feeding induced a much higher incidence of HCC in male mice with substantially increased intrahepatic retention of hydrophobic BAs and decreased hepatic expression of tumor-suppressive microRNAs. Metagenomic analysis showed differences in gut microbiota involved in BA metabolism between normal male and female mice, and such differences were amplified when mice of both sexes were exposed to STZ-HFD. Treating STZ-HFD male mice with 2% cholestyramine led to significant improvement of hepatic BA retention, tumor-suppressive microRNA expressions, microbial gut communities, and prevention of HCC. Additionally the sex-dependent differences in BA profiles in the murine model can be correlated to the differential BA profiles between men and women during the development of HCC. These results uncover distinct male and female profiles for gut microbiota, BAs, and microRNAs that may contribute to sex-based disparity in liver carcinogenesis, and suggest new possibilities for preventing and controlling human obesity-related gastrointestinal cancers that often exhibit sex differences.

Epidemiological studies have provided compelling evidence for the role of sex in liver cancer etiology and survival. A United States 2016 cancer statistic report stated that, the incidence of newly diagnosed hepatic cancer was 28,410 for male and 10,820 cases for females, a 2.6:1 ratio for male vs. female. The number of deaths from hepatic cancer in 2016 was 18,280 for males and 8,890 for females, a 2.1:1 ratio for male vs. female^1^. Hepatocellular carcinoma (HCC) accounts for 90% of primary liver cancer^2^. A study conducted over a 17 year period for a cohort of 1,138 individuals with HCC (32% female and 68% male) revealed that in patients matched for tumor burden and residual liver function, survival, defined as the time between date of diagnosis and date of death, was longer for women^3^.

Sex-based outcomes for HCC extend to other mammals; murine models in which carcinogens such as N,N-diethylnitrosamine induced HCC also revealed a similar or higher susceptibility to HCC for males^4^. Sex hormones have been shown to contribute to the sex bias and the commonly accepted view is that estrogens are protective while androgens stimulate hepatocellular carcinogenesis^5^. Castration, administration of either estrogens or anti-androgen agents as well as genetic ablation of the androgen receptor in hepatocytes, limits HCC development in male rodents^6^. Conversely, ovariectomy, testosterone supplementation or genetic inactivation of estrogen receptor alpha increases HCC development in female mice^5^,^7^. It has also been observed that the hormonal influence on the sexual dimorphism of HCC is sensitive to environmental influences^8^. Sex-specific enterotypes may influence metabolism of hormones, including estrogens^9^ and testosterone^10^. Recently the bacterial species, *Clostridium scindens*, has been shown to convert glucocorticoids into androgens, implicating the gut microbiota as another source of androgens in addition to the host endocrine system^11^. Antibiotic-induced depletion of the intestinal microbiota in mice suppressed development of HCC, thus revealing a role for microbiota in HCC^12^. These results support the hypothesis that sex-dependent enterotypes influence liver carcinogenesis.

Recently, several studies have demonstrated that bile acids (BAs) are closely associated with human liver diseases such as fatty liver disease, cirrhosis, and HCC^13^. Four BAs, cholic acid (CA), chenodeoxycholic acid (CDCA), deoxycholic acid (DCA) and lithocholic acid (LCA) have been shown to be hepatotoxic when present in concentrations ranging from 0.3% (CA, CDCA) to 0.1% (DCA) and finally, 0.03% (LCA) whereas ursodeoxycholic acid (UDCA), at levels as high as 3% showed no hepatotoxic effects as evidenced by alanine aminotransferase (ALT) levels^14^. Abnormal BA profiles are often associated with altered gut microbiota since BA metabolism is largely carried out in the intestine via bacterial bile salt hydrolase (BSH) enzymes^15^. We and others have shown that several BAs, including DCA, LCA, CDCA, and taurochenodeoxycholate (TCDCA), have cytotoxic and cancer-promoting properties^16^,^17^. Our studies also showed that there were significant differences of serum BA profiles between males and females^18^.

MicroRNAs (miRNAs) have been previously employed as useful biomarkers for cancer diagnosis and prognosis. miRNA transcription as well as cholesterol and BA homeostasis has been shown to be regulated by the farnesoid X receptor (FXR) and small heterodimer partner (SHP) nuclear receptors^19^. We previously observed differences in hepatic miRNA expression patterns, including miR-321, miR-26a, miR-10b, miR-125b-1, miR-99b, miR-325, miR-342, and miR-129-2 in liver tissue between men and women with HCC^20^. Mounting evidence suggests a close association between BAs and miRNAs in the regulation of hepatic and whole-body metabolic homeostasis^21^. For example, the expression of miR-22, a key tumor-suppressive miRNA, is regulated by BAs^22^. In addition, miR-29a expression is suppressed in the liver of both CCl_4_ and common bile-duct ligation murine models^23^. Its promoter activity was significantly increased by FXR-binding through a likely FXR-responsive element^24^.

Based on these findings, we hypothesized that sex differences in gut microbiota, BA metabolism, and miRNA expression contribute to differential risks of liver carcinogenesis between males and females. To test this hypothesis, we utilized a NASH-HCC C57BL/6 J murine model, which is highly relevant to human liver disease progression from steatosis, NASH, fibrosis to HCC induced through a streptozotocin-high fat diet (STZ-HFD)^25^,^26^. All male mice in the model group developed whereas the incidence in female mice was only 1 out of 8 throughout the experimental period. We investigated differential fecal microbiota profiles, BA, and miRNA contributing to hepatocellular carcinogenesis in a sex-specific manner. The results obtained in this study provide critical insights into sex-specific metabolic phenotypes associated with carcinogenesis. The hypothesis was further verified in human male and female individuals in the development of HCC.

## Results

### The incidence of STZ-HFD induced HCC is significantly higher in male mice than in female mice

**Figure 1:**
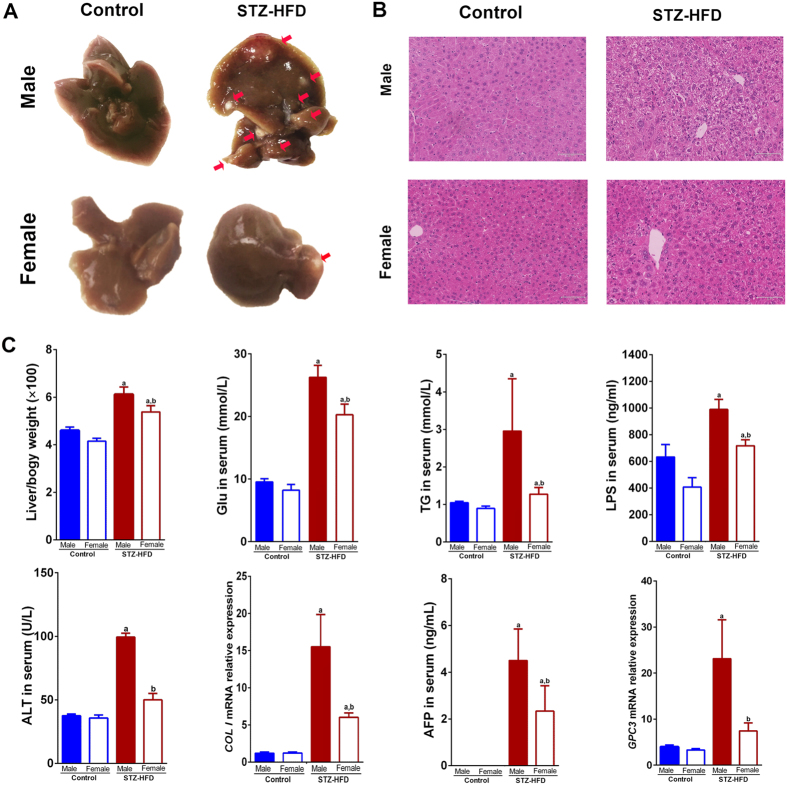
Pathophysiological features of NASH–HCC model mice. (**A**) Representative macroscopic photographs of mouse livers. Arrowheads indicate HCCs; (**B**) H&E stained liver sections from control and STZ-HFD mice from males and females. Bar = 72 μm; (**C**) Bar plots of liver to body weight ratio, serum levels of fasting glucose, TG, LPS, ALT and AFP, and mRNA levels of Col I and Gpc-3 in liver. a, p < 0.05, compared to controls; b, p < 0.05, females compared to males.

STZ-primed neonatal mice fed with HFD resulted in HCC at week 20. In 100% of the male mice (n = 8), HCC liver tumors were observed (Fig. 1, arrowhead). However, we observed that 1 out of 8 female mice developed liver tumors and the number of tumors in the single female mouse was significantly lower than those found in male mice (Fig. 1A and B). Regardless of sex, liver to body weight ratio, fasting serum glucose, serum triglyceride (TG), serum lipopolysaccharide (LPS), ALT, alpha-fetoprotein (AFP), and mRNA expression of Collagen type I (Col I) and Glypican-3 (Gpc-3) were significantly higher in STZ-HFD-exposed mice than the controls (Fig. 1C). When grouped by sex, no significant differences were observed in controls whereas male mice that underwent STZ-HFD intervention had statistically significant higher liver to body weight ratio, fasting serum glucose, serum TG, serum LPS, ALT, AFP, mRNA levels of Gpc-3 and Col I relative to females. Based on our results, the incidence of HCC in male STZ-HFD mice was 100% vs. a 12.5% HCC incidence observed in female STZ-HFD mice, thus revealing a clear sex disparity for development of HCC.

### STZ-HFD intervention induced significant alteration in gut microbiota

**Figure 2:**
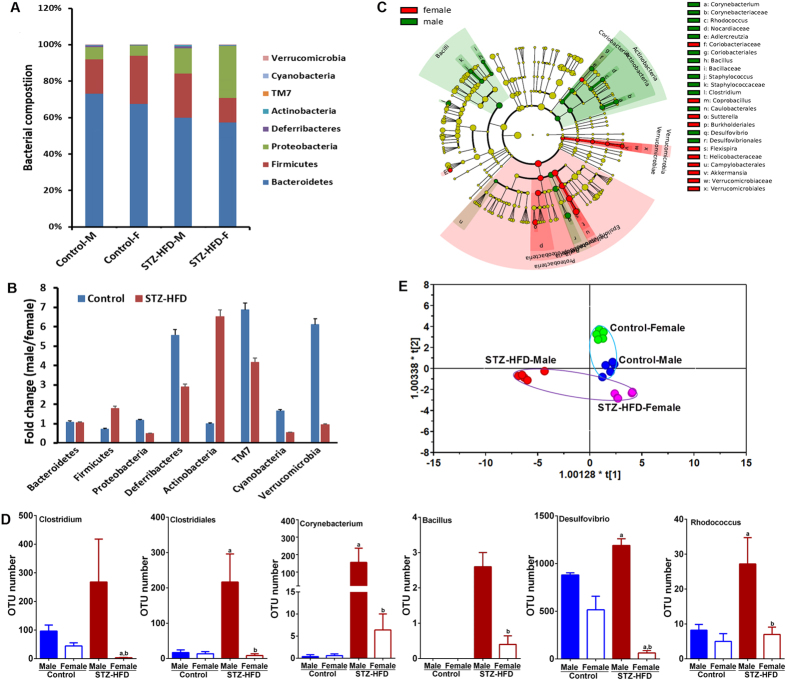
(**A**) Bar charts summarizing overall microbial composition of control (n = 5) and STZ-HFD mice (n = 5) at phylum level. (**B**) Fold change of gut microbiota at the phylum level in males relative to females in both control group and STZ-HFD group. (**C**) Taxonomic representation of statistical differences in relative abundances between STZ-HFD-exposed female and male mice. Linear discriminant analysis Effect Size (LEfSe) was conducted on relative taxonomic abundances from phylum until genus level. Differences are represented in the colour of the most abundant class (red: female, green: male, yellow: non-significant (p < 0.05)). Each circle’s diameter is proportional to the taxon’s abundance. (**D**) Bar charts of representative gut microbiota involved in BA metabolism with significant change due to STZ-HFD intervention. ^a,b^p < 0.05, model vs. normal or normal male vs. normal female or model male vs model female (Mean ± SE). (**E**) OPLS-DA scores plot (R2X = 0.766, R2Y = 0.957, Q2 = 0.721) of mouse gut mcirobiota profiles involved in BA metabolism for classification by sex and STZ-HFD treatment.

To monitor shifts in the composition of fecal microbiota in the development of HCC, Illumina MiSeq sequencing was performed. In total, 969585 valid sequences were generated and a total of 639057 reads (average of 31953 ± 3692 S.D. reads per sample) were obtained for 20 samples (n = 5 in each group) after quality control. A total of 1159 operational taxonomic units (OTUs) were then identified by grouping reads at the 97% similarity level. The Shannon and Chao1 indices all reached stable values as indicated by the observed plateaus seen in for each group (Supplementary Fig. S1A,B). This indicated that most of the bacterial richness, ie., the number of taxa (species) present in a sample at a particular phylogenetic level (Chao1 index) and diversity, ie., a metric that combines both richness and the evenness of abundance of different taxa (Shannon index) in these communities were covered (Supplementary Fig. S1A,B). The Rarefaction curves revealed that although new rare phylotypes would be expected with additional sequencing, most of the diversity had already been captured as each curve has started to plateau (Supplementary Fig. S1C). Compared with the controls, the STZ-HFD group exhibited lower alpha-diversity as indicated by Chao1 (t test, P = 0.005), ACE (t test, P = 0.006) and Shannon (t test, P = 0.14) for both males and females (Supplementary Table S1). The Simpson (t test, P = 0.04) index is also a measure of diversity and was also significantly different between STZ-HFD mice and controls but the interpretation of this index with respect to our data is that control mice had a slightly higher value indicating more dominance from one taxa relative to the STZ-HFD groups. This was confirmed at the level of phylum in Fig. 2A. ACE or Chao1 were significantly different between control and STZ-HFD group in female mice but with no significant difference in male mice, highlighting sex differences in community richness with STZ-HFD female mice showing lower community richness relative to males. This result is also evident from the rarefaction curve (Supplementary Fig. S1C). In contrast, a significant difference in the Simpson index was only observed between control and STZ-HFD male mice (Supplementary Table S1). All of the indices describing microbiota α-diversity were found be significant when comparing STZ-HFD male vs. female mice. Female STZ-HFD mice scored lower in both diversity and richness relative to the male STZ-HFD mice. These results highlight the sex specific shifts in gut microbiota that occurred upon STZ-HFD treatment.

At the phylum level, the majority of the bacterial phyla identified in the fecal samples were encompassed by Bacteriodetes (73.1% in control male mice and 67.4% in control female mice, 59.9% in STZ-HFD male mice and 57.3% in STZ-HFD female mice, on average) and Firmicutes (18.9% in control male mice and 26.5% in control female mice, 24.3% in STZ-HFD male mice and 13.5% in STZ-HFD female mice, on average) as depicted in Fig. 2A. This is also reflected by the relatively high Simpson index (Supplementary Table S1).

The relative amounts measured for other bacteria were; (1) Proteobacteria (6.7% in control male mice and 5.7% in control female mice, 13.9% in STZ-HFD male mice and 28.5% in STZ-HFD female mice, on average), (2) Deferribacteres (1.0% in control male mice and 0.2% in control female mice, 1.0% in STZ-HFD male mice and 0.3% in STZ-HFD female mice, on average), and (3) Actinobacteria (0.1% in control male mice and 0.1% in control female mice, 0.7% in STZ-HFD male mice and 0.1% in STZ-HFD female mice, on average). Twenty weeks of HFD feeding induced widespread changes in gut microbial community structure at the phylum level, with abundances of Proteobacteria increased and abundances of Bacteroidetes decreased in all mice. Interestingly, Firmicutes were decreased significantly after 20 weeks of HFD feeding in female mice, in contrast to a significantly increased Firmicutes population in male mice. The ratio of Firmicutes to Bacteroidetes was markedly increased upon HFD in male mice (0.26 to 0.41) and decreased significantly in female mice (0.39 to 0.24). Verrucomicrobia was significantly decreased in male mice but was increased in female mice. As shown in Fig. 2B, differences in gut microbiota at the phylum level were observed between males and females in the controls and the difference remained after STZ-HFD intervention.

### Identification of bacterial taxa abundances associated with STZ-HFD intervention and sex

Microbial compositions of STZ-HFD in male and female mice were compared by applying the linear discriminant analysis (LDA) effect size (LEfSe) algorithm on relative taxonomic abundances at different phylogenetic levels (from phylum until genus level). When compared to controls (Supplementary Fig. S2A,B), STZ-HFD mice showed decreased abundance of *Coriobacteriaceae, Bacteroidaceae, Paraprevotellaceae, Prevotella, Lactobacillus, Lactobacillaceae, Anaerostipes, Coprobacillus*, and *Erysipelotrichaceae*. On the other hand, *Corynebacterium, Corynebacteriaceae, Rhodococcus, Nocardiaceae, Streptophyta, Bacillus, Bacillaceae, Staphyiococcus, Aerococcus, Enterococcus, Allobaculum, Erysipelotrichales, Klebsiella, Acinetobacter, Pseudomonadales, Enterobacteriales* and *Turicibacteraies* were significantly increased in STZ-HFD-exposed mice, compared to control mice, based on the alpha-values for the factorial Kruskal-Wallis test between groups (p < 0.05) and the logarithmic LDA score (>2.0). Next, sex-dependent differences in taxa were identified by directly comparing STZ-HFD exposed males with STZ-HFD-exposed females (Fig. 2C and Supplementary Fig. S2C). This revealed a higher abundance of *Corynebacterium, Corynebacteriaceae, Rhodococcus, Nocardiaceae, Adlercreutzia* which belong to the phylum Actinobacteria, *Bacillus, Bacillaceae, Staphylococcus, and Staphylococcaceae* within the class of *Bacilli, Desulfovibrio* and *Desulvibrionales* within the phylum of Proteobacteria, and *Clostrodium* within the phylum of Firmicutes in male mice when compared to female mice. In particular, we observed that the bacteria involved in BA metabolism were different between males and females and became significantly different after STZ-HFD intervention (Fig. 2D).

As revealed by the OPLS-DA scores plot established using gut microbiota involved in BA metabolism (R2X = 766, R2Y = 0.957, Q2(cum) = 0.721), the control male, control female and STZ-HFD female mice were located in the first and second quadrant while STZ-HFD male mice were located at the fourth quadrant away from the controls (Fig. 2E).

We also performed the MANOVA on the first three weighted microbial PCoA axes and found that the influence of STZ-HFD intervention (p < 0.0001), sex (p = 0.001) and the interaction of STZ-HFD intervention and sex were significant (p < 0.0001) per the Wilks’ test. Thus far we have established; (1) that male mice are more susceptible to HCC, (2) that there are significant sex disparities in gut microbiota in STZ-HFD treated mice, (3) significant differences at the phylum level exist between male and females both in control and STZ-HFD mice, (4) significant differences in BA metabolizing microbiota were present in male vs. female mice for both control and STZ-HFD groups.

### STZ-HFD resulted in significantly higher levels of hepatic BAs in male mice than in female mice

**Figure 3:**
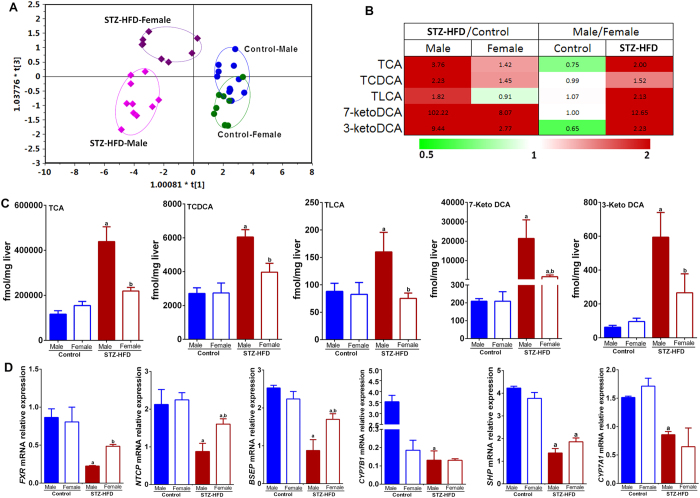
Hepatic BA profiles are significantly different between males and females upon STZ-HFD exposure. (**A**) OPLS-DA scores plot (R2X = 0.801, R2Y = 0.739, Q2 = 0.607) of mouse hepatic BA profiles for classification by sex and STZ-HFD treatment. (**B**) Heatmap showing the fold change values of mean concentration of BAs for STZ-HFD model group compared to control group in males and females and for males compared to females in control group and model group. (**C**) Bar plots of representative BAs with significant change due to STZ-HFD intervention. ^a,b^p < 0.05, model vs. normal or normal male vs. normal female or model male vs model female (Mean ± SE). (**D**) The mRNA expression of genes in normal group and STZ-HFD intervention group with quantitative real-time polymerase chain reaction (qRT-PCR) analysis in male and female mice. ^a^p < 0.05, compared to controls; ^b^p < 0.05, females compared to males.

Given the significant sex-associated differences in BA metabolizing microbiota, we next investigated the hepatic BA profiles in the mice. STZ-HFD treatment led to significantly altered liver BA concentrations in both sexes (Fig. 3A) as revealed by the OPLS-DA scores plot established using hepatic BA data (R2X = 0.801, R2Y = 0.739, Q2(cum) = 0.607). The hepatic BAs, 3-ketodeoxycholic acid (3-ketoDCA), taurocholic acid (TCA), taurolithocholic acid (TLCA), taurochenodeoxycholic acid (TCDCA), and 7-ketodeoxycholic acid (7-ketoDCA), were significantly increased in STZ-HFD-exposed mice compared to controls (Fig. 3 and Supplementary Fig. S3). More substantial increases in hepatic BA levels were observed in male STZ-HFD mice. Moreover, the increase in TLCA was only observed in males exposed to STZ-HFD and decreased levels of TLCA was observed, but with no statistical significance, in females (Fig. 3B). Among the significantly altered liver BAs, TCA, TCDCA, TLCA, 7-ketoDCA and 3-ketoDCA were significantly higher in males than in females after STZ-HFD intervention (Fig. 3C).

STZ-HFD also led to significant increases in fecal and serum BA levels. Fecal BAs, TDCA, GLCA, GDCA, and GCA were increased in male STZ-HFD mice relative to control. The results were more variable for female mice with TDCA, GDCA and GCA showing increases with STZ-HFD and GLCA slightly but significantly decreased in the model vs. control (Supplementary Fig. S3). GDCA, TDCA, and GLCA, secondary, microbiota metabolized BAs were significantly higher in male relative to female model mice (Supplementary Fig. S3).

Serum concentrations of TCDCA, TCA, ACA, TLCA, 3-ketoDCA, and 7-ketoDCA were lower in control male vs. female mice (Supplementary Fig. S4). Notably, serum levels of TCDCA, ACA, 3-ketoDCA and 7-ketoDCA were found to be significantly higher in STZ-HFD treated male relative to female mice. This flip from low to high concentration of specific BAs in male relative to female model mice reminds us of the flip in abundance discussed earlier for the Firmicutes/Bacteriodetes ratio which showed the ratio to go from low in control to high in STZ-HFD males and *vice versa* in female mice. Both of these results indicate sex specific changes upon STZ-HFD treatment. In order to determine whether BA transport into and out of the liver was affected by STZ-HFD and was responsible for the greater increase in hepatic BAs for STZ-HFD male mice, we next examined mRNA expression for the BA transporter genes.

### Sex disparity was found in the expression of hepatic BA transporter mRNA

A qRT-PCR analysis revealed that genes involved in hepatic BA transport and synthesis were significantly different between sexes. In STZ-HFD treated male mice, hepatic FXR expression was significantly decreased. In STZ-HFD female mice FXR showed a decreasing trend that was not statistically significant. FXR is known to regulate the SHP and thus, accompanying the decrease in FXR mRNA expression was a decrease in mRNA expression for SHP for both male and female STZ-HFD mice. A depressed expression of FXR mRNA could also explain decreased expression of mRNA for BA transporters. The expression of mRNA for the major BA uptake transporter, the sodium-taurocholate cotransporting polypeptide (NTCP), was suppressed by STZ-HFD treatment (Fig. 3D). In addition, the bile salt export pump (BSEP) mRNA was found to be significantly decreased in male model mice relative to control. The female model mice exhibited BSEP mRNA levels that were significantly decreased relative to control but significantly increased with respect to male model results. Thus, these alterations in BA transport may lead to increased BA accumulation in hepatocytes and BA-induced liver injury. The expression of mRNA for BA synthesis, CYP7A1 and CYP7B1, was sinificantly down-regulated after STZ-HFD intervention in male STZ-HFD mice relative to control but the smaller decrease observed for female STZ-HFD mice was not statistically significant. Notably, in female mice, no significant difference in the mRNA expression of hepatic SHP, CYP7A1, and CYP7B1 was found between model and normal mice (Fig. 3D).

The mRNA expression of FXR, CYP7B1, BSEP and SHP, was lower and expression of NTCP and CYP7A1 were higher in normal female mice when compared to normal male mice. The expression of the above-mentioned genes was less altered in female mice than in male mice when exposed to STZ-HFD (Fig. 3D).

### Hepatic expression of miRNAs was significantly different between STZ-HFD treated male and female mice

**Figure 4:**
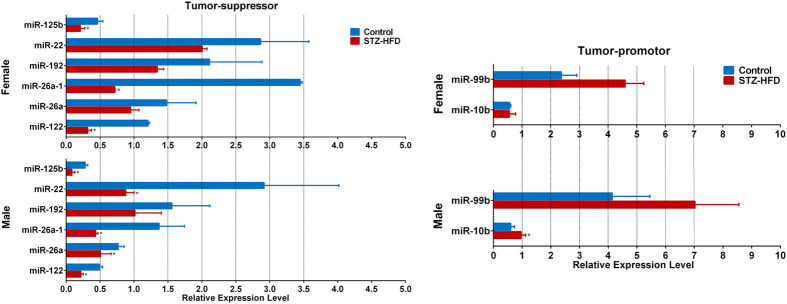
Hepatic expression of tumor suppressive miRNAs, miR-26a, miR-26a-1, miR-192, miR-122, miR-22 and miR-125b, and tumor promoting miRNAs, miR-10b and miR-99b in NASH-HCC model male and female mice. Data are presented as the mean ± S.E. *p < 0.05, model compared to control.

Since the expression of miRNAs are different between men and women with HCC and can be regulated by BAs^20^,^21^,^22^, we further analyzed miRNAs in liver tissues of male and female mice from the STZ-HFD model group and control group. As shown in Fig. 4, the tumor suppressive miRNAs, miR-26a, miR-26a-1, miR-192, miR-122, miR-22, and miR-125b were lower, whereas the tumor-promoting miRNAs, miR-10b and miR-99b were higher in males than in females in both the STZ-HFD group and the control group. As expected, the expression of tumor-suppressive miRNAs were decreased whereas the tumor-promoting miRNAs were increased much more in male mice than in female mice after STZ-HFD treatment, which presumably facilitated the development of liver tumors in male model mice.

### BA-binding resin treatment can prevent HCC in male mice with recovered levels of differentially expressed BAs, gut microbiota and miRNAs

**Figure 5:**
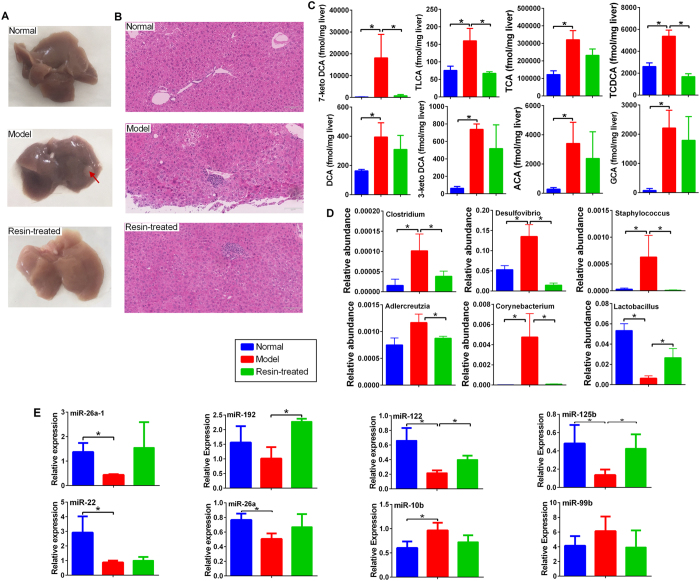
(**A**) Representative macroscopic photographs of livers. Arrowheads indicate HCC. (**B**) H&E stained liver sections from normal, NASH-HCC and NASH-HCC-cholestyramine mice at week 20. Bar = 90 μm. (**C**) Levels of BAs significantly increased in NASH-HCC mice were attenuated after cholestyramine treatment. (**D**) Relative abundances of altered *Clostridium, Desulfovibrio, Staphylococcus, Adlercreutzia, Corynebacterium* and *Lactobacillius* were normalized after cholestyramine treatment. (**E**) Expression of altered miRNAs, miR-26a, miR-26a-1, miR-192, miR-122, miR-22, miR-125b, miR-10b and miR-99b were normalized after cholestyramine treatment.

The levels of BAs including TCA, TCDCA, TLCA, 3-keto DCA, and 7-keto DCA, and the gut microbiota including *Corynebacterium, Corynebacteriaceae, Rhodococcus, Nocardiaceae, Adlercreutzia, Bacillus, Bacillaceae, Staphylococcus, Staphylococcaceae, Lactobacillales, Desulfovibrio, Desulvibrionales, Clostrodium,* and *Clostridiales*, were much higher in male STZ-HFD mice than in female STZ-HFD mice. The miRNAs were also significantly different between males and females. In a separate study using the STZ-HFD mice model we used a BA sequestrant, cholestyramine, to remove the intestinal BAs in male mice. We observed that depletion of secondary BAs in the intestine by cholestyramine prevented the STZ-HFD male mice from developing tumors, none in the cholestyramine treatment group (n = 8) developed tumor while all of the mice in the model group (n = 8) developed liver tumors (Fig. 5A and B). After cholestyramine administration, the levels of BAs, TCA, TCDCA, TLCA, 3-keto DCA and 7-keto DCA, were significantly decreased in the liver (Fig. 5C). The abnormal gut microbial profile and miRNAs were also normalized with cholestyramine intervention (Fig. 5D and E).

### The BA metabolic profiles were significantly different between men and women

**Figure 6:**
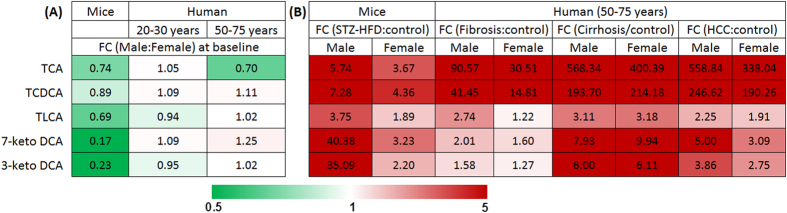
Heatmap showing (**A**) the fold change (FC) values of mean concentration of BAs between male and female in mice as well as in human subjects at baseline; and (**B**) the FC values of mean concentration of BAs in liver disease subjects relative to controls in males and females. Shades of red and green represent fold increase and fold decrease of a BA, respectively, in males relative to females at baseline or in liver disease subjects relative to controls (see color scale).

Results from our recently published data^18^ showed that the serum BA levels including TCA, TCDCA, TLCA, 7-keto DCA, 3-keto DCA, DCA and GCA were significantly different between healthy men and women, similar to the mice data (Supplementary Fig. S4). To verify the findings from the animal studies that differentially expressed BAs impact liver carcinogenesis in a sex dependent manner, we profiled the serum BAs in age and BMI matched liver disease patients and healthy participants of men and women. Serum BA measurement in liver fibrosis (n = 30, 15 males and 15 females aged 50–75 years), cirrhosis (n = 40, 20 males and 20 females aged 50–75 years), and HCC (n = 40, 30 males and 10 females aged 50–75 years) patients and healthy participants (n = 40, 20 males and 20 females aged 50–75 years) showed that the levels of BAs differentially expressed between healthy men and women were significantly increased in patients (both sexes) but with higher fold changes in men than in women in the development of liver disease (Fig. 6 and Supplementary Fig. S4).

## Discussion

**Figure 7:**
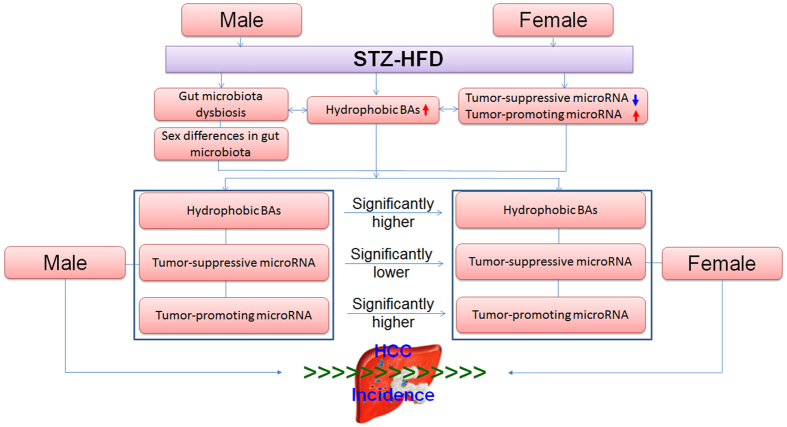
Proposed mechanisms involved in sex differences in liver carcinogenesis.

The metabolic defects in the liver-BA-microbiota axis may serve as an intrinsic link between gut microbiota and obesity-related liver carcinogenesis. We focused on the characterization of the differential microbiota compositions, hepatic BAs, and miRNA expressions in a well-characterized STZ-HFD induced HCC murine model, to investigate how BAs and microbiota are associated with hepatocellular carcinogenesis in a sex-specific manner (Fig. 7). We decided to use the STZ-HFD induced NASH-HCC model because it is highly relevant to human liver disease that progresses from steatosis, NASH, fibrosis to HCC^25^,^26^. The model is fast and HCC-specific, where all male mice develop HCC within 20 weeks. The chemically induced, such as diethylnitrosamine, HCC mouse model typically takes about 40 weeks to develop not only liver tumors but also others such as gastric, skin, respiratory and haematopoietic^27^.

Altered gut microbiota associated with an altered BA profile is a common etiology for nonalcoholic steatohepatitis, liver cirrhosis and gastrointestinal cancer^28^. Our results showed that the gut microbiota is significantly different between normal male and female mice. Such differences in microbiota lead to different BA synthesis, metabolism, and transport in liver between the males and females, and become pathologically significant when exposed to STZ-HFD.

Loss of microbiota diversity, the alpha diversity, appears as the most constant finding of intestinal dysbiosis^29^. It has been reported that fecal samples from colon cancer patients had less bacterial diversity compared with samples from healthy individuals and a lower amount of bacterial diversity in the gut may indicate a lack of balance in the complex bacterial population^30^. Findings also showed that the non-obese patients with nonalcoholic fatty liver disease were characterized by a decrease in gut microbial diversity^31^. As shown in our data, a significant decrease in the Simpson index (alpha diversity) was only observed in STZ-HFD male mice relative to controls (Supplementary Table S1), which may be a reason why the male mice had a higher risk of developing HCC relative to females.

Gut microbiota alterations characterized by a significant elevation in aerobic and pro-inflammatory *Enterobacter, Enterococcus*, and *Clostridium* species and a reduction in beneficial anti-inflammatory *Bifidobacterium* and *Lactobacillus* are often found in liver disorder patients^15^. One recent report performed a metagenomic study on fecal samples from liver cirrhosis patients with or without HCC and the result showed that although there was no significant differences on the fecal counts of *Enterobacteriaceae, Enterococcus* species, *Bifidobacterium* species, *Bacteroides* species, *Lactobacillus* species and *Clostridium* species between them^32^, but the levels of *Escherichia coli* was significantly higher in HCC patients, which was believed to be able to deconjugate conjugated BAs to form secondary BAs^15^,^33^. The conversion of primary to secondary BAs and de-conjugation of BAs into free BAs are also attributed to bacteria *Clostridium, Eubacterium, Bifidobacterium* and *Lactobacillus*. The significantly decreased abundance of *Lactobacillus* and *Lactobacillaceae* and significantly increased abundance of *Enterococcus, Erysipelotrichales*, and *Enterobacteriales* found in STZ-HFD treated mice is consistent with the observation of abnormally high BA levels in the liver. In addition, male mice had higher abundances of *Lactobacillales, Clostrodium* and *Erysipelotrichaceae*, which may have led to the significantly increased levels of hepatic BAs, particularly, the hydrophobic and cytotoxic BAs, TCA, 7-ketoDCA, TLCA and TCDCA, compared to female mice. Oral administration of a BA sequestrant effectively prevented tumorigenesis in the male mice with normalized levels of the differential BAs and gut microbial species identified between male and female mice, suggesting a novel HCC preventive strategy by counteracting the pro-carcinogenic toxicity of intestinal BAs. Rats treated with diethylnitrosamine was associated with a significant suppression of *Lactobacillus* species, *Bifidobacterium* species and *Enterococcus* species as well as intestinal inflammation while probiotics administration to those diethylnitrosamine treated rats showed decreased liver tumor growth, highlighting the importance of gut homeostasis in the pathogenesis of HCC^34^.

An important secondary BA, DCA, was shown to be carcinogenic in mice in 1940^35^. A recent study revealed that HFD altered the gut microbiota in a murine model, and this resulted in an increased hepatic level of DCA^36^. 7-keto DCA, a microbial product from CA like DCA^37^, was found at high levels in male HCC mice. The higher abundance of *Lactobacillales, Clostrodium* and *Erysipelotrichaceae* rich with high BSH activity in intestine together with significantly increased 7-keto DCA levels in the liver of the male mice was closely associated with higher incidence of HCC. Other hepatic BAs, particularly, TCA, TCDCA, and TDCA have all been previously implicated as etiologic agents in cancer of gastrointestinal tract, including cancer of esophagus, stomach, small intestine, liver, biliary tract, pancreas and colon/rectum^38^.

We also observed that tumor-suppressive miRNAs, miR-26a, miR-26a-1, miR-192, miR-122, miR-22, and miR-125b were significantly decreased in STZ-HFD mice compared to controls with significantly lower levels in males than in females. It was reported that FXR can regulate miRNA transcription^19^ to exert its protective effect in the gastrointestinal tract^22^. The significantly higher miR-26a, miR-26a-1 and miR-122 levels in females may be attributed to the observed higher levels of FXR in female model mice compared to males.

Inflammation is known to stimulate cell death and increase cell turnover, thus promoting liver tumorigenesis. As expected, STZ intervention and continuous HFD stimulates oxidative stress and inflammation, as evidenced by increased levels of ALT, and AFP and Gpc-3, significant markers for HCC, as shown in other reports^25^,^39^. LPS has been implicated as an important cofactor in the pathogenesis of liver injury and has been shown to promote hepatic fibrosis^40^. In the pathogenesis of chronic inflammation and autoimmune diseases, dysregulated intestinal BAs may be a causal factor for increased absorption of bacterial LPS^41^, thereby promoting systemic inflammation in the organism. Levels of Gpc-3, AFP, LPS and ALT were significantly lower in female mice when compared to male mice, providing another possible reason for the higher incidence of liver tumor in male mice.

In addition, bile flow in hepatic inflammation is reduced and is correlated with loss of gene expression and lower protein levels for FXR and the NTCP transporter. Decreased hepatic transporter function combined with reduction in FXR signaling leads to enhanced BA sequestration in liver, causing sustained inflammation that can progress to HCC. It has been shown that FXR function and expression is decreased to 40% of normal level at stage I HCC and decreases further with progressive later stages^42^. Furthermore, it is known that *Fxr*^*−/−*^ *Shp*^*−/−*^ mice develop spontaneous liver tumors when exposed to chronically elevated BAs^43^. We observed a sex difference in the expression of genes involved in BA transport and synthesis, including the higher levels of FXR, BSEP and NTCP in female mice with STZ-HFD exposure, compared to male mice. As previously reported, expression of NTCP was shown to be expressed at higher levels in female murine models^44^. In HCC, NTCP is down regulated in comparison to the surrounding healthy tissue. This is supported by the finding that NTCP expression is absent in the hepatoma cell line HepG2^45^, and inhibition of NTCP leads to an increase of serum BA levels^46^. It was reported that severe forms of BSEP deficiency syndrome patients are at significant risk to develop HCC^47^. The decreased mRNA expression of FXR, BSEP, SHP and NTCP may translate into corresponding lower protein levels which could cause decreased BA pump rate from liver to bile and increased reabsorption of BAs from the portal vein. This may explain the observed accumulation of BAs detected in the liver of the STZ-HFD mice in this study. Therefore, the observed sex difference in FXR, BSEP, NTCP, CYP7A1 and CYP7B1 gene expressions may result in changes in enterohepatic circulation/BA synthesis and the differential accumulation of cytotoxic BAs in hepatocytes, thus contributing to a higher liver cancer incidence in male mice.

It has been reported that sex hormones contributed to the sex bias and estrogens are protective while androgens stimulate hepatocellular carcinogenesis^5^. However, we did not measure the sex hormone levels in the studied mice in our study and this is a limitation.

In summary, BA and gut microbiota influence each other and jointly regulate various signaling pathways to maintain the health of the digestive tract. Under normal circumstances, luminal BA levels rise to sufficient concentrations to form micelles, which facilitate lipid emulsification and absorption. Pathology develops when the gut microbiota is altered. Understanding such bidirectional communication between BAs and microbiota in the gut-liver axis may provide important insights into mechanisms of liver carcinogenesis. Our results uncovered microbiota and BAs associated with liver cancer in both sexes while providing a mechanistic link between gut bacteria and host liver pathology in a sex-dependent manner. This work will also provide important directions for future usage of diet and probiotics for liver cancer prevention and control in a sex-specific manner.

## Methods

### STZ-HFD induced NASH-HCC mouse model

All mouse procedures were performed as described in the Supplementary Information. All animal procedures were performed in accordance with the “Guide for the Care and Use of Laboratory Animals” prepared by the National Academy of Sciences and published by the National Institutes of Health (NIH publication 86–23, revised 1985). The experimental protocol was approved by the Institutional Animal Committee of Shanghai University of Traditional Chinese Medicine, Shanghai, China. Serum, liver, and feces samples were collected for biochemical, histological, BA quantitation and Illumina MiSeq sequencing (see Supplementary Information for details).

### Fibrosis, cirrhosis and HCC patients and controls

Fasting serum samples were collected from patients diagnosed with fibrosis (n = 30), cirrhosis (n = 40), and HCC (n = 40) and healthy controls (n = 110) recruited at Shuguang Hospital affiliated to Shanghai University of Traditional Chinese Medicine (Shanghai, China), and Xiamen Hospital of Traditional Chinese Medicine (Xiamen, China) from April 2013 to December 2013.

The study was approved by the institutional human subjects review board of the Shanghai University of Traditional Chinese Medicine and Xiamen Hospital of Traditional Chinese Medicine. All participants signed informed consent forms for the study. All methods were carried out in accordance with the approved guidelines. Detailed information is provided in the Supplementary Information.

### miRNA assay

Detailed information is provided in the Supplementary Information.

### Statistical analysis

All statistical analyses were calculated using GraphPad Prism (version 6.0; GraphPad Software, San Diego, USA) and SPSS 22.0 (IBM SPSS, USA). Data are expressed as mean ± SEM. To test differences between the groups in biochemical measurements for statistical significance, normally distributed data were analyzed by tests with the Holm-Sidak method for multiple comparisons correction. Data that did not meet the assumptions of analysis were analyzed by the Mann-Whitney U test. We regarded *p* values of <0.05 as significant. Detailed information is provided in the Supplementary Methods.

## Additional Information

**How to cite this article:** Xie, G. *et al*. Sex-dependent effects on gut microbiota regulate hepatic carcinogenic outcomes. *Sci. Rep.* **7**, 45232; doi: 10.1038/srep45232 (2017).

**Publisher's note:** Springer Nature remains neutral with regard to jurisdictional claims in published maps and institutional affiliations.

## Supplementary Material

Supplementary Information
