# Supplementary material for: Sex-dependent effects on gut microbiota regulate hepatic carcinogenic outcomes
Source: Sci Rep. 2017 Mar 27;7:45232. doi: 10.1038/srep45232 (PMC5366919; doi:10.1038/srep45232)
Supplement: Supplementary Information [file srep45232-s1.pdf]

# Supporting Information

# Sex-dependent effects on gut microbiota regulate hepatic carcinogenic outcomes

Guoxiang Xie, Xiaoning Wang, Aihua Zhao, Jingyu Yan, Wenlian Chen, Runqiu Jiang, Junfang Ji, Fengjie Huang, Yunjing Zhang, Sha Lei, Kun Ge, Xiaojiao Zheng, Cynthia Rajani, Rosanna Alegado, Jiajian Liu, Ping Liu, Jeremy Nicholson, Wei Jia

## Supporting Methods

***STZ-HFD induced NASH-HCC mouse model and experimental design. Experimental 1****:* New born male and female C57BL/6J mice purchased from Vital River Laboratory Animal Technology Co. Ltd. (Beijing, China) were divided into four groups: control group (male and female) and NASH-HCC model group (male and female). Control mice were housed without any treatment and fed normal diet (composed of 12 kcal% fat). The NASH-HCC mice were subjected to a single subcutaneous injection of 200 µg STZ (Sigma, MO, USA) 2 days after birth and feeding with HFD (composed of 60 kcal% fat) *ad libitum* after 4 weeks of age for 16 weeks to induce the HCC.[1] The body weight of all animals were measured and recorded once a week. At week 20, 8 mice in each group were euthanized and their livers were removed and stored at −80 °C for histological and lipid content analysis, including hematoxylin–eosin (H&E) staining and triglyceride (TG)

analysis. Fasting blood glucose was measured using an automatic biochemical analyzer (Hitachi 7180, Tokyo, Japan). Before sacrifice, fecal samples were collected from each mouse. All stool samples were stored at −80 °C prior to 16S rRNA gene sequencing.

***Experimental 2***: We found that the BA levels were significantly higher in male mice than in female mice with significantly higher incidence in hepatic carcinogenesis in Experimental 1; we repeated the NASH-HCC mice model to see whether the BA-binding resin, cholestyramine, can attenuate/prevent liver carcinogenesis in male mice. Three groups of mice were included: (1) control male; (2) model (STZ-HFD) male, and (3) STZ-HFD-BA resin male group, mice were fed with HFD diet containing 2% cholestyramine. All the procedures are the same as in Experimental 1. At week 20, 8 mice in each group were euthanized and liver, plasma, and fecal samples were collected.

All animal procedures were performed in accordance with the ‘‘Guide for the Care and Use of Laboratory Animals’’ prepared by the National Academy of Sciences and published by the National Institutes of Health (NIH publication 86–23, revised 1985). The experimental protocol was approved by the Institutional Animal Committee of Shanghai University of Traditional Chinese Medicine, Shanghai, China.

***Liver histological analysis*.** Liver tissues were fixed in 10% neutral-buffered formalin, embedded in paraffin blocks, and processed by routine H&E staining. The stained sections were subsequently examined for histopathological changes.

***Measurement of TG in the liver.*** The levels of triglyceride (TG) in the liver was measured using Elisa kits from BlueGene Biotech, Shanghai, China.

***LPS analysis in blood serum.*** Serum lipopolysaccharides (LPS) concentrations were determined using a mouse LPS Elisa kit (BlueGene Biotech, Shanghai, China) according to the manufacturer's protocol.

***AFP measurement in blood serum.*** Serum alpha-fetoprotein (AFP) was measured using TBA-40FR Fully Automatic Biochemical Analyzer (Toshiba, Japan).

***Real-time quantitative polymerase chain reaction.*** Total RNA was isolated from liver tissue using TRIzol reagent (Invitrogen, Shanghai, China) according to the manufacturer's protocol. RNA concentration was determined by a GE Nanovue Ultramicro spectrophotometer (Healthcare Bio-Sciences AB, USA). Expression of the target mRNA was measured in triplicate by the comparative cycle threshold method on the ViiA™ 7 Real-Time PCR System (Invitrogen, Life Technologies,) using SYBR Green PCR kit. The forward and reverse primers of target mRNA for real-time PCR were designed and synthesized by Sangon Biotech (Shanghai, China) and sequences are shown in Supporting Information Table 2. Target gene expression was normalized to β-actin levels and the relative expression of the target genes was calculated using the "dCT" a.k.a comparative Ct approach.

***Fibrosis, cirrhosis and HCC patients and controls.*** Age and BMI matched patients diagnosed with fibrosis (n=30, 15 males and 15 females aged 50-75 years), cirrhosis (n=40, 20 males and females aged 50-75 years), and HCC (n=40, 30 males and 10 females aged 50-75 years) were recruited at Shuguang Hospital

affiliated to Shanghai University of Traditional Chinese Medicine (Shanghai, China), and Xiamen Hospital of Traditional Chinese Medicine (Xiamen, China) from April 2013 to December 2013. *All patients were clinically stable at the time of assessment* and received a liver biopsy directed by ultrasonography within 1 week after inclusion in the study except those diagnosed as having decompensated cirrhosis.

Two groups of age and BMI matched healthy men and women participants were recruited as healthy controls from the Physical Examination Center of Shuguang Hospital: one group of 35 men and 35 women aged 20-30 years and the second group of 20 men and 20 women aged 50-75 years. Routine biochemical tests were done to ensure that the study subjects were free of inflammation and metabolic diseases. B-mode ultrasound examination was done to exclude participants with fatty liver.

The study was approved by the institutional human subjects review board of the Shanghai University of Traditional Chinese Medicine and Xiamen Hospital of Traditional Chinese Medicine. All methods were carried out in accordance with the approved guidelines. All participants signed informed consent forms for the study.

Fasting serum samples were collected for BA quantitation.

***BA quantitation.*** The BA levels in liver, serum, and feces were quantitatively measured using a Waters ACQUITY ultra performance liquid chromatography coupled with a Waters XEVO TQ-S mass spectrometer with an ESI source (UPLC-TQMS, Waters, Milford, MA) according to our previously reported protocol.[2]

For liver samples, liver tissue was precisely weighed (~50 mg) and homogenized in 75 μl ice-cold 50% methanol, and then centrifuged at 20,000 g for 10 min. The supernatant was transferred to a clean tube. Then 80 μL of ice-cold methanol: chloroform (3:1, v/v) were added to the remaining pellet and homogenized again. After recentrifugation, the two supernatants were combined, spiked with 10 μL of the internal standards (IS, 100 nM for GCA-d4, TCA-d4, TCDCA-d9, UDCA-d4, CA-d4, GCDCA-d4, GDCA-d4, DCA-d4, and 200 nM for LCA-d4) and vacuum dried. The extracts were reconstituted with 40 μL acetonitrile (with 0.1% formic acid) and 40 μL water (with 0.1% formic acid). After centrifugation, 5 μL of the supernatants were injected into the UPLC-TQMS for measurement.

For fecal samples, the stool samples were precisely weighed (~50 mg) and homogenized in 200 μl ice-cold 50% methanol, and then centrifuged at 20,000 g for 10 min. The supernatant was transferred to a clean tube. Then 200 μL of ice-cold methanol: chloroform (3:1, v/v) were added to the remaining pellet and homogenized again. After centrifugation, the two supernatants were combined, spiked with 10 μL of IS and vacuum dried. The extracts were reconstituted with 50 μL acetonitrile (with 0.1% formic acid) and 50 μL of water (with 0.1% formic acid). After centrifugation, 5 μL of the supernatants were injected into the UPLC-TQMS for measurement.

For serum samples, 50 μL aliquots of the serum samples were mixed with 150 μL of methanol containing internal standards (IS, 0.10 μM of CA-D4, DCA-D4, GCA-D4 and LCA-D4), and then incubated for 10 min at room temperature. After

centrifugation at 20,000 g for 10 min, the supernatant was transferred to a clean tube, vacuum dried and reconstituted with 40 μL acetonitrile (with 0.1% formic acid) and 40 μL water (with 0.1% formic acid). After recentrifugation, the supernatant was used for UPLC-TQMS measurement.

All separations were performed with an ACQUITY BEH C18 column (1.7 μm, 100 mm × 2.1 mm) (Waters, Milford, MA). BA raw data obtained with negative mode detection were analyzed using TargetLynx applications manager version 4.1 (Waters, Milford, MA) to obtain calibration equations and the quantitative concentration for each BA in the samples.

***Illumina MiSeq sequencing*.** 16S rRNA gene polymerase chain reaction amplification, Illumina MiSeq sequencing, and data analysis were performed by Personal Biotech Co., Ltd. (Shanghai, China). Fecal DNA was extracted using a QIAamp stool DNA mini kit (Qiagen, 51504) following the manufacturer's protocol. Pyrobest DNA Polymerase (Ta-KaRa, DR500A) was used for the amplification of the V4–V5 hypervariable regions of 16S rRNA gene from microbial genome DNA which were harvested from fecal samples using the universal primers (forward primers: 5' GTGCCAGCMGCCGCGG 3' and reverse primers: 5' CCGTCAATTCMTTTRAGTTT 3'). The PCR condition was as follows: initial denaturation at 98°C for 5 min; 98°C denaturation for 30 sec, 50°C annealing for 30 s, and 72°C extension for 30 s, repeated for 25 cycles; final extension at 72°C for 5 min. PCR product was excised from a 1.5% agarose gel and purified by Axy-Prep DNA Gel Extraction Kit (Axygen, AP-GX-500). V4–V5 amplicons were sequenced using pair-end method by Illumina Miseq with a 6 cycle index read.

The average length of sequence reads was 221 bp. Sequence reads were trimmed so that the average Phred quality score for each read was above 25 and its length was more than 50 bp; after trimming, these reads were assembled by Flash software (http://www.genomics.jhu.edu/software/FLASH/index.shtml) and the reads which could not be assembled were discarded. Only those reads with consecutive and identical base shorter than 6 bp, without ambiguous bases, were used for further analysis. The pyrosequencing chimeras were discarded using the Uchime filtering in the Mothur pipeline (Version 1.31.2).[3] Sequences clustering was performed by UCLUST (QIIME) with a similarity cutoff of 97 %, and clustered into operational taxonomic units (OTUs). The longest sequence in each cluster was chosen to be the representative sequences, which were annotated by RDP-classifier 2.2 (QIIME).

Rarefaction was applied to the OTUs so that the number of reads per sample would be identical and subsequently used to perform alpha and beta diversity calculations. Alpha diversity was assessed using the metrics Chao1, Shannon, Simpson, ACE and Observed species.

Principal coordinate analysis and distance based redundancy analysis (db-RDA) were performed to explain the (dis)similarity in species composition (using weighted UniFrac distances) using the following explanatory variables: treatment and gender, using the add-in XLSTAT (version 2015.5.01, Addinsoft, New York, USA) developed for Microsoft Excel. MANOVA were also performed on the leading weighted microbial PCoA axes to determine whether any of these exhibit the effects of STZ-HFD intervention, gender or gender*STZ-HFD intervention using

the add-in XLSTAT. To identify differential abundant taxa from pyrosequencing data, the linear discriminant analysis effect size (LEfSe) method was applied on taxonomic read abundances.[4] Both treatment and gender were used as classification in this analysis.

***miRNA assay*.** Total RNAs were extracted from liver tissue using standard TRIZOL (Invitrogen, Carlsbad, CA) methods. The expression of mature miRNAs was measured using Taqman MicroRNA Assays specific for miR-10b, miR-99b, miR-342, miR-22, miR-192, miR-125b, miR-129, miR-26a, miR-26a-1, miR-26a2, and miR-26b after reverse transcription (Thermo Fisher Scientific, Waltham, MA). The Taqman MicroRNA Assay for U6 RNA was used to normalize the relative abundance of miRNAs. The experiments were performed in triplicate.

***Statistical analysis.*** Multivariate statistical analysis, orthogonal partial least squares-discriminant analysis (OPLS-DA) was conducted to get an overview of the BA profiles among the different groups using SIMCA-P+ 14.0 (Umetrics, Umeå, Sweden). All other statistical analyses were calculated using GraphPad Prism (version 6.0; GraphPad Software, San Diego, USA) and SPSS 22.0 (IBM SPSS, USA). Data are expressed as mean ± SEM. To test difference between the groups in biochemical measurements for statistical significance, normally distributed data were analyzed by tests with the Holm-Sidak method for multiple comparisons correction. Data that did not meet the assumptions of analysis were analyzed by the Mann-Whitney U test. We regarded $p$ values of $< 0.05$ as significant.

## References

[1] Fujii M, Shibazaki Y, Wakamatsu K, Honda Y, Kawauchi Y, Suzuki K, et al. A murine model for non-alcoholic steatohepatitis showing evidence of association between diabetes and hepatocellular carcinoma. Med Mol Morphol 2013;46:141-152.
[2] Xie G, Zhong W, Li H, Li Q, Qiu Y, Zheng X, et al. Alteration of bile acid metabolism in the rat induced by chronic ethanol consumption. FASEB journal : official publication of the Federation of American Societies for Experimental Biology 2013;27:3583-3593.
[3] Edgar RC, Haas BJ, Clemente JC, Quince C, Knight R. UCHIME improves sensitivity and speed of chimera detection. Bioinformatics 2011;27:2194-2200.
[4] Segata N, Izard J, Waldron L, Gevers D, Miropolsky L, Garrett WS, et al. Metagenomic biomarker discovery and explanation. Genome biology 2011;12:R60.

**Table S1**. Comparison of diversity indices between the gut microbiota of controls (C) and model mice (STZ-HFD); values are shown as mean ± standard deviation.

| | STZ-HFD | C | STZ-HFD (male) | C (male) | STZ-HFD (female) | C (female) | P1 | P2 | P3 | P4 | P5 |
|---|---|---|---|---|---|---|---|---|---|---|---|
| Shannon index | 4.83±0.20 | 5.17±0.10 | 5.17±0.19 | 5.25±0.05 | 4.49±0.28 | 5.09±0.2 | 0.141 | 0.661 | 0.225 | 0.462 | 0.056 |
| Simpson's index | 0.90±0.01 | 0.93±0.01 | 0.92±0.01 | 0.94±0.01 | 0.88±0.01 | 0.92±0.01 | .036 | 0.0262 | 0.140 | 0.184 | 0.0123 |
| ACE | 581.63±24.52 | 661.35±8.15 | 644.05±15.03 | 655.43±12.36 | 519.2±23.04 | 667.28±11.35 | 0.006 | 0.556 | 0.005 | 0.611 | 0.0006 |
| Chao1 | 579.8±24.75 | 663.7±8.63 | 641.02±14.67 | 658.93±15.1 | 518.58±25.86 | 668.46±9.81 | 0.005 | 0.411 | 0.0098 | 0.6740 | 0.0150 |

P1: model vs. control; P2: model male vs. control male; p3: model female vs. control female; p4: control male: control female; p5: model male vs. model female

**Table S2.** Primer sets for quantitative RT-PCR analysis.

| Gene | Full name | Primer sequences (5'——3') | |
|---|---|---|---|
| FXR | Farnesoid X receptor | Forward | GCTGTGTGTTGTCTGTGGAGA |
| | | Reverse | GGCGTTCTTGGTAATGCTTC |
| OATP1A1 | Organic anion transporting polypeptide 1a1 | Forward | GCATTGGGTCATCAGGAAAC |
| | | Reverse | ATGGCTGCGAGTGAGAAGAT |
| SHP | small heterodimer partner | Forward | TTCCTTGCTTTGGATACAGT |
| | | Reverse | GAGGTTTGGGGAGTCATCA |
| GPC3 | Glypican 3 | Forward | CGGTGGTTAGCCAGATCATTGAC |
| | | Reverse | AGAACTTTACCCTTGGGCACAGAC |
| CYP7A1 | Cytochrome P450 7A1 | Forward | CTGGGCTGTGCTCTGAAGT |
| | | Reverse | GGGAGTTTGTGATGAAGTGGA |
| CYP7B1 | Cytochrome P450 7B1 | Forward | TGCCGTCTCCTTTAGGTACA |
| | | Reverse | GCTTCTCTGCCACACTTTCA |
| BSEP | bile salt export pump | Forward | AGCAGGCTCAGCTGCATGAC |
| | | Reverse | AATGGCCCGAGCAATAGCAA |
| NTCP | sodium-dependent taurocholate cotransporting polypeptide | Forward | TCTCTGCTCTCTTCCGACTA |
| | | Reverse | GTGACATTGAGGATGGTAGA |
| ASBT | Apical sodium dependent bile acid transporter | Forward | GGTACAGGTGCCGAACAGTAG |
| | | Reverse | AGATGAGTGGGAAGGTGAACA |
| ACTB | Beta-actin | Forward | 5'-CTT TGC AGC TCC TTC GTT GC-3' |
| | | Reverse | 5'-CCT TCT GAC CCA TTC CCA CC-3' |

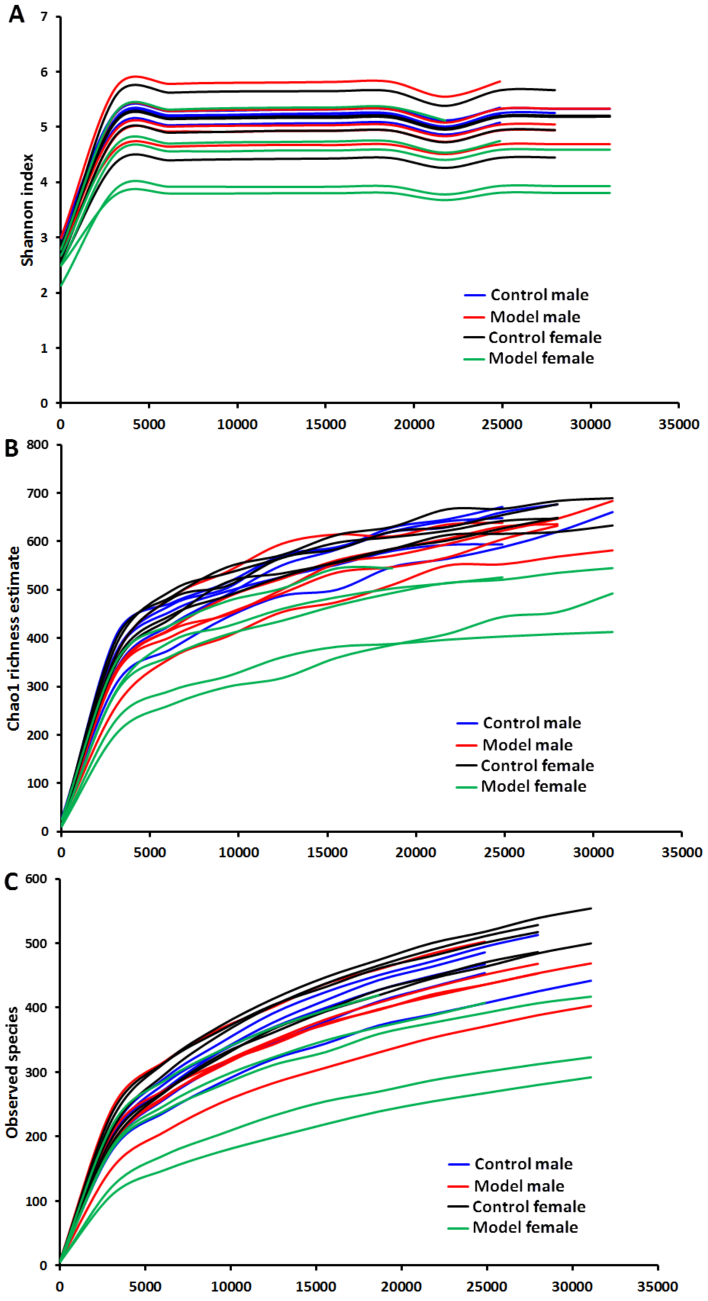


**Fig. S1.** Evaluation of the sequencing depth in each sample. (A) Shannon diversity index curves of the samples. (B) Chao 1 richness estimates. (C) Rarefaction curves of the samples.

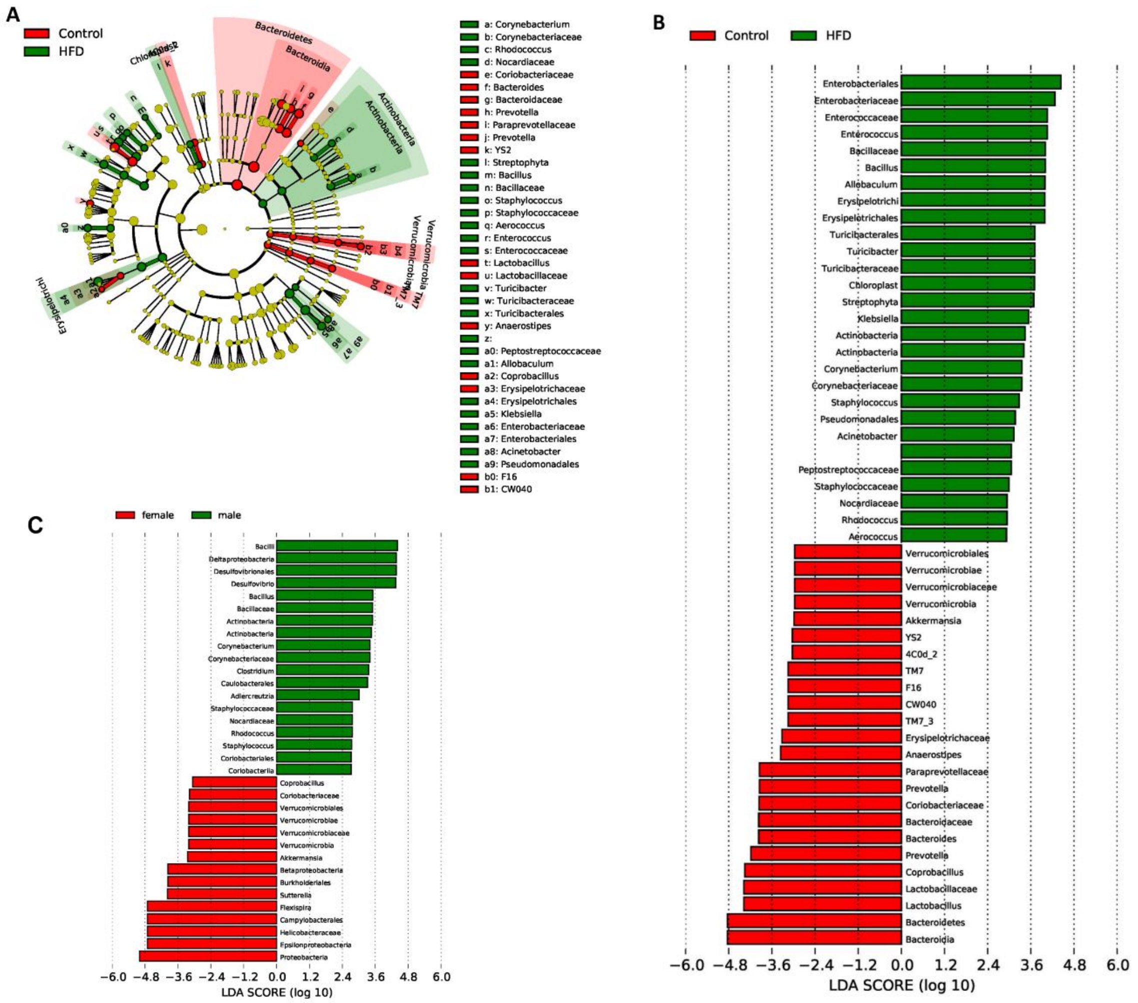


**Fig. S2**. (A) Linear discriminant analysis Effect Size (LEfSe) was conducted on relative taxonomic abundances from phylum until genus level. Differences are represented in the color of the most abundant class (red: control or STZ-HFD-female, green: STZ-HFD or STZ-HFD-male, yellow: non-significant ($p<0.05$)). Each circle's diameter is proportional to the taxon's abundance. (B) Taxonomic representation of statistical differences in relative abundances between control and STZ-HFD-exposed mice or (C) STZ-HFD-exposed female and male mice.

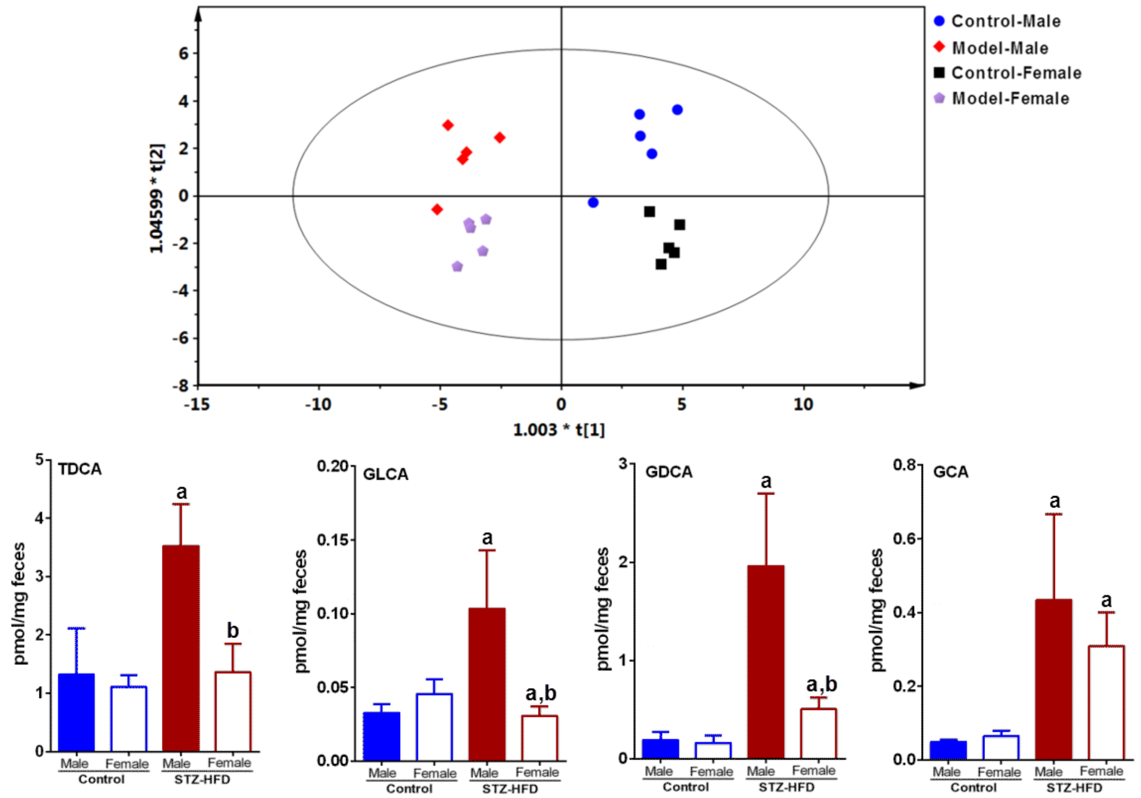


**Fig. S3.** Fecal BA profiles were significantly different between males and females upon STZ-HFD exposure. [a], $p < 0.05$, compared to controls; [b], $p < 0.05$, females compared to males (Mean ± SE).

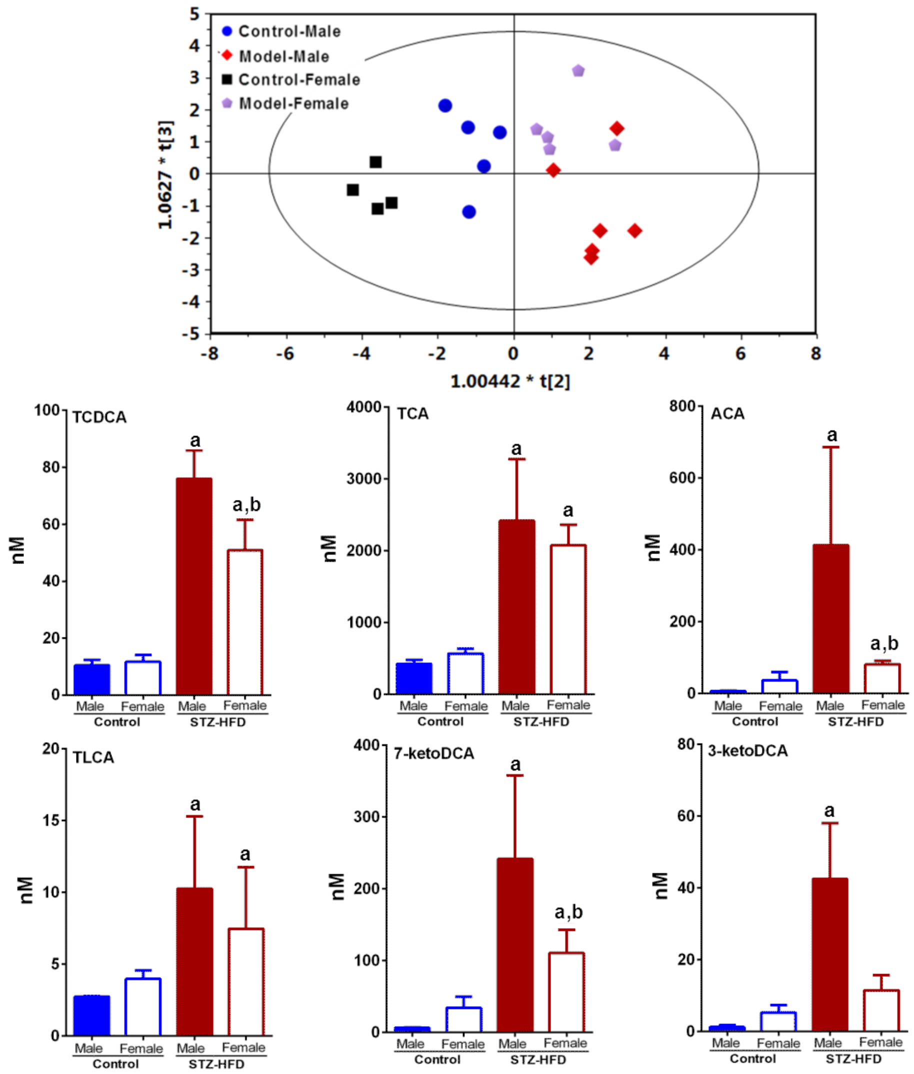


**Fig. S4.** Serum BA profiles are significantly different between males and females upon STZ-HFD exposure. [a], $p < 0.05$, compared to controls; [b], $p < 0.05$, females compared to males (Mean ± SE).
